# CryoEM in the fast lane of structural biology

**DOI:** 10.1107/S2052252526007220

**Published:** 2026-07-23

**Authors:** Richard Henderson, S. Samar Hasnain

**Affiliations:** a MRC Laboratory of Molecular Biology, Francis Crick Avenue CambridgeCB2 0QH United Kingdom; b Department of Biochemistry, Cell and Systems Biology, Institute of Systems, Molecular and Integrative Biology University of Liverpool LiverpoolL69 7ZB United Kingdom

**Keywords:** cryoEM, structural biology, atomic resolution, membrane proteins, metalloenzymes, multiprotein complexes

## Abstract

Single-particle cryoEM has made visualization of biological molecules and their complexes at high resolution possible without the need for crystallization. The last decade has seen a rapid growth in the number of structures determined by cryoEM; for membrane proteins, the total number of cryoEM structures has exceeded that determined by crystallography.

It is nearly seventy years since the first of the three-dimensional structures of two haem-containing proteins, myoglobin and haemoglobin, appeared (Kendrew *et al.*, 1958 ▸, 1960 ▸; Perutz *et al.*, 1960 ▸), launching the field of structural biology and transforming the approach to understanding biological systems and mechanism. These structures were determined by X-ray crystallography using a rotating-anode source, some 20 years before the emergence of the first dedicated storage-ring-based crystallographic facilities. The high intensity and brilliance of X-ray beams from synchrotron sources together with community-based software efforts, *e.g.CCP4* (Agirre *et al.*, 2023 ▸) and *Phenix* (Liebschner *et al.*, 2019 ▸), transformed X-ray crystallography to become a mainstream endeavour engaging the wider scientific community rather than the restricted pursuit of a few specialists in dedicated crystallographic laboratories. This democratization of crystallographic techniques brought major successes and scientific breakthroughs (for a review, see the front cover of the January 2025 issue of the *Journal of Synchrotron Radiation* and Hasnain, 2025 ▸).

It took 50 years from the first crystallographic structures of proteins for single-particle cryoEM (Kühlbrandt, 2014 ▸; Liao *et al.*, 2013 ▸) to move from ‘Blobology’ to high-resolution structures of macromolecules and large multiprotein complexes, fulfilling many of the theoretical predictions made in 1995 (Henderson, 1995 ▸) by overcoming some of the key barriers, including the development of direct electron detectors (McMullan *et al.*, 2014 ▸) and improved computer programs for single-particle image analysis. Fundamental breakthroughs came from the development of a plunge-freezing method for preparing thin films of amorphous ice on electron microscope grids together with the introduction of a vacuum specimen environment in the electron microscope, resulting in high-quality images of biological structures from ferritin to adenovirus (Dubochet *et al.*, 1982 ▸, 1984 ▸, 1988 ▸). These developments have revolutionized cryoEM as a mainstream structural biology tool (Henderson, 2018 ▸; Frank, 2018 ▸; Dubochet, 2018 ▸), prompting the International Union of Crystallography to launch a dedicated section on cryoEM in its premier journal *IUCrJ* (Subramaniam *et al.*, 2016 ▸) and to devote a dedicated session entitled ‘Method of the Decade – CryoEM’ at its 26th Congress and General Assembly in Hyderabad in 2017 (Hasnain, 2016 ▸).

We celebrate the start of the second decade of the ‘resolution revolution’ and the 10th anniversary of the dedicated cryoEM section in *IUCrJ* by bringing together some of the leading figures and broader community in the special issue at https://journals.iucr.org/special_issues/2026/cryoem/ to provide an overview of the field and a forward look at how the resolution revolution is continuing, with cryoEM not only reaching parity with crystallography but being now in the ‘fast lane of structural biology’. This is clearly demonstrated for membrane proteins (Table 1 ▸), where the number of structures determined by single-particle cryoEM now significantly exceeds that determined by X-ray crystallography. It is also evident from this table that more than 350 of these structures have been determined to high resolution (2 to 2.5 Å). Owing to the rapidly growing impact of cryoEM, we are beginning to catch up on the coverage of structures of membrane proteins, which has so far significantly lagged behind the structural coverage of soluble proteins. The number of membrane-protein structures has more than doubled in the last five-year period (2020–2024) compared with the previous five years, with a 50% increase in the number of structures in the high-resolution window of 2 to 2.5 Å.

The higher resolution is often important for understanding many biological mechanisms. In metalloenzymes, where the stereochemistry around metals and metal clusters is often critical for the mechanism, atomic resolution structures are highly desirable, where ‘atomic resolution’ is defined as that at which carbon–carbon bonds are resolved and is typically <1.2 Å. We have nice examples emerging of high-resolution cryoEM structures (1.7–1.9 Å) of three monofunctional catalase enzymes in our laboratory (Slowik & Henderson, unpublished). There are now a few cryoEM structures at atomic resolution (<1.2 Å), although all of them are of apoferritin (Yip *et al.*, 2020 ▸; Maki-Yonekura *et al.*, 2023 ▸; Küçükoğlu *et al.*, 2024 ▸; Nakane *et al.*, 2020 ▸) (Table 2 ▸). In the next resolution shell (1.2–1.4 Å), we have eight cryoEM structures, but only two of them are not apoferritin structures – DPS (DNA protection during starvation protein) (Montemayor *et al.*, 2025 ▸) and rubisco (Croy *et al.*, 2025 ▸). We can expect many more atomic resolution cryoEM structures in the coming decade, particularly with improvements in 100–200 keV microscopes, improved detectors, further reduction in beam-induced specimen movement, reproducible grid preparations and improvement in analysis software packages that harness the benefits of AI (Vinothkumar & Henderson, 2016 ▸; Cheng, 2018 ▸). Despite these advances and the potential they offer for studying smaller and smaller particles, we still anticipate the trend to be weighted towards larger assemblies. ▸

CryoEM has also been transforming the structural biology landscape of multiprotein complexes, including mammalian mitochondrial super-complex CIII_2_CIV (Vercellino & Sazanov, 2021 ▸) and mammalian respiratory super-complex I_1_III_2_IV_1_ (Wu *et al.*, 2016 ▸; Nakano *et al.*, 2026 ▸). The prowess of cryoEM for larger assemblies is clearly demonstrated by Fig. 2 ▸, where nearly 50% of the cryoEM structures are >280 kDa structures. In contrast, 70% of the X-ray structures are <80 kDa structures.

The papers presented in the special issue at https://journals.iucr.org/special_issues/2026/cryoem/ show glimpses of the developments that are on the horizon, including improved computational approaches that disentangle diverse conformations (Noone & Bubeck, 2026 ▸); the use of a cold field emission gun in a 200 keV microscope, a high-resolution objective lens polepiece and an energy filter to provide 1.24 Å resolution structures (Danev *et al.*, 2026 ▸); a method for generating regularized reprojections directly from the noisy particles that does not rely on explicit 3D density reconstruction (Van *et al.*, 2026 ▸); and improved processing of movies (Burton Smith & Murata, 2026 ▸). There are several papers that cover a wide range of applications and systems, including drug discovery (Wang *et al.*, 2026 ▸).

Before concluding, we would like to reiterate discussion of use of the term cryoEM. When cryogenic sample stages were introduced into electron microscopes by the biological community, the term ‘cryo’ was added to the abbreviation EM and it has been universally adopted to stand for cryo electron microscopy or electron cryo microscopy. As we have pointed out in this journal before, neither the electrons nor the microscopes are cryogenically cooled (Henderson & Hasnain, 2023 ▸). Perhaps in the coming decade cryoEM will more accurately be taken as meaning *cryogenic-sample Electron Microscopy*.

## Figures and Tables

**Figure 1:**
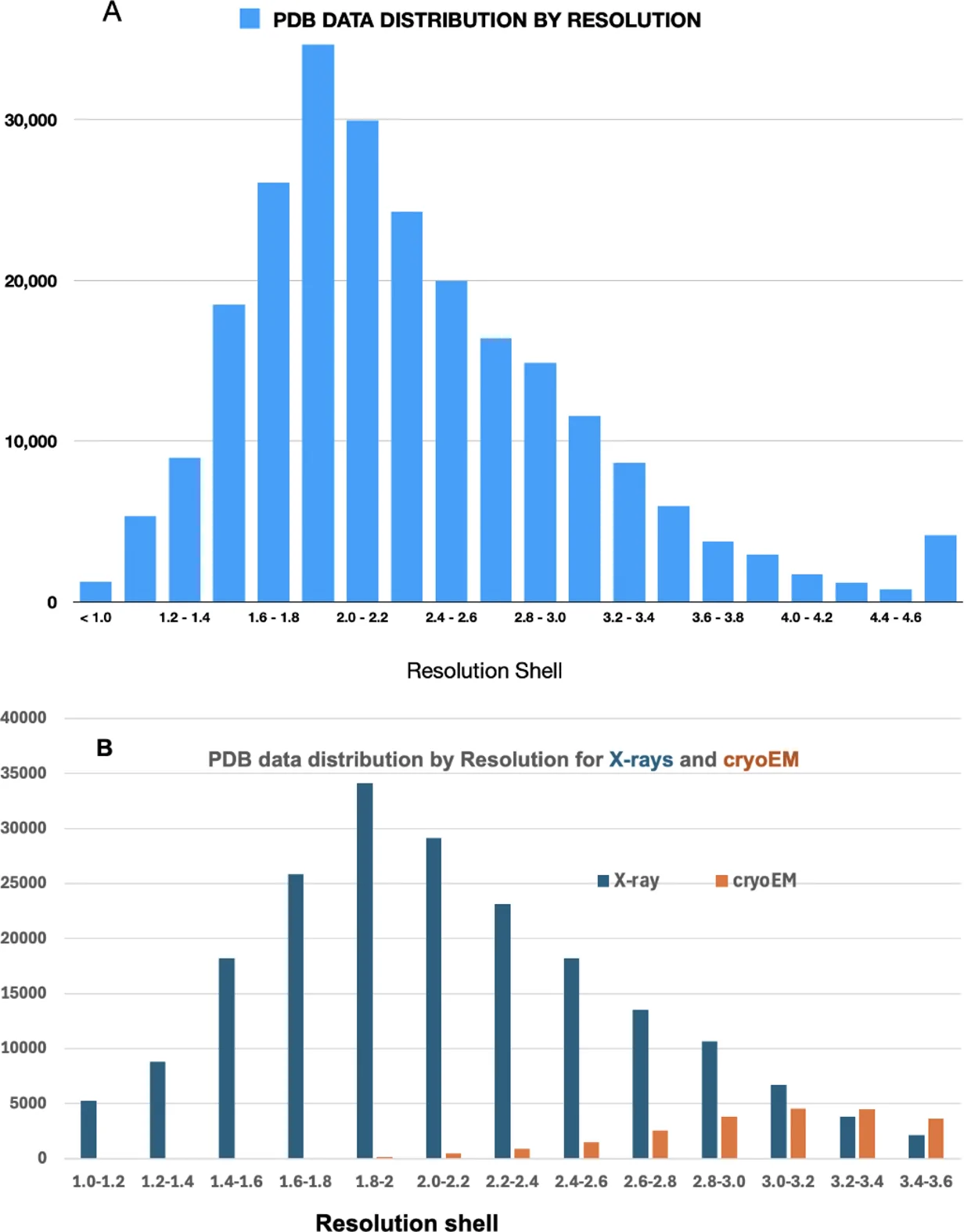
(A) Distribution of structures in the PDB with resolution, with X-ray structures dominating the high-resolution window of 1.8–2.0 Å while cryoEM exhibits a maximum number of structures in the 3.0–3.2 Å resolution window (B), see also Table 2 ▸.

**Figure 2:**
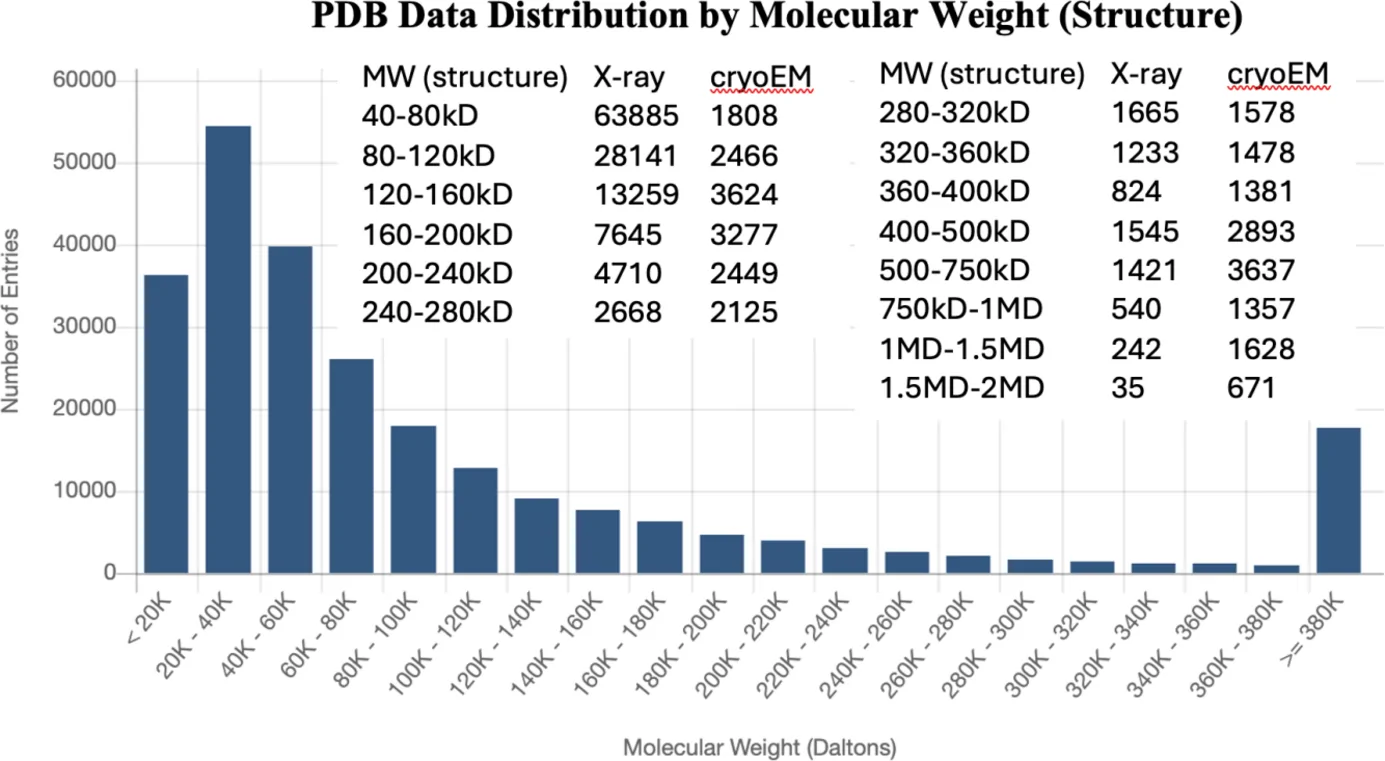
Illustrative representation of the structures by molecular weight.

**Table 1:** Numbers of structures of membrane proteins in the PDB (April 2026)

		Resolution		
Period	No. of structures	2–2.5 Å	2.5–3.0 Å	3.0–3.5 Å
1975–1984	2	0	0	1
1985–1989	4	1	1	0
1990–1994	58	10	6	7
1995–1999	244	60	43	29
2000–2004	571	139	116	51
2005–2009	1034	236	190	148
2010–2014	1729	293	386	368
2015–2019	2983	412	595	627
2020–2024	7679	646	1941	2694
2025–4/2026	1258	78	355	506
				
cryoEM	8386	363	1979	3160
X-ray	6170	1508	1647	1254

**Table 2:** Numbers of structures in different resolution ranges in the PDB that have been determined by X-ray crystallography and cryoEM

Resolution	X-ray	cryoEM
1.0–1.2 Å	5266	3†
1.2–1.4 Å	8819	8†
1.4–1.6 Å	18222	13
1.6–1.8 Å	25806	50
1.8–2 Å	34117	179
2.0–2.2 Å	29158	448
2.2–2.4 Å	23129	855
2.4–2.6 Å	18190	1499
2.6–2.8 Å	13537	2531
2.8–3.0 Å	10683	3816
3.0–3.2 Å	6695	4550
3.2–3.4 Å	3836	4492
3.4–3.6 Å	2123	3651

† All of the cryoEM structures apart from two in the 1–1.2 Å and 1.2–1.4 Å resolution ranges are those of ferritin.
